# Genome drafting of nosocomial infection CRE *Klebsiella pneumoniae* confirming resistance to colistin and eravacycline, carrying *bla*_NDM-1_, *mcr-1*, and *bla*_KPC-2_, in neonatology from November to December 2023

**DOI:** 10.3389/fcimb.2024.1528017

**Published:** 2025-01-27

**Authors:** Xinlong Zhou, Kailash Wagh, Guizhen Lv, Devender Sharma, Wei Lei

**Affiliations:** ^1^ Dongguan Maternal and Child Health Care Hospital, Dongguan, Guangdong, China; ^2^ Department of Microbiology, Dr Ulhas Patil Medical College, Jalgaon, India; ^3^ Dongguan Molecular Diagnostic Technology and Infectious Disease Medical Test Engineering Research Center, Dongguan Labway Clinical Laboratory Co., Ltd., Dongguan, China; ^4^ Department of Microbiology, Himachal School of Dental Medical Sciences, Mandi, India

**Keywords:** carbapenem-resistant *Klebsiella pneumoniae*, neonatology, antibiotic resistance, whole-genome sequencing, nosocomial infections

## Abstract

**Background:**

Carbapenem-resistant *Klebsiella pneumoniae* (CRKP) is a critical pathogen in healthcare settings, associated with high mortality due to its extensive antibiotic resistance. In this study, we report an outbreak of CRKP in a neonatal intensive care unit (NICU) within a 200-bed tertiary hospital. The main goal of this study was to characterize the phenotypic and genomic profiles of the CRKP isolates involved in the outbreak and to gain insights into their resistance mechanisms and transmission dynamics within the NICU.

**Methods:**

The study was conducted between November and December 2023 in a 5-bed NICU. Monthly surveillance cultures were performed to monitor colonization and infection with multidrug-resistant organisms. CRKP isolates were obtained from blood and nasal swabs of affected neonates. Identification and antimicrobial susceptibility testing were initially conducted using the Vitek^®^2 system with an N-395 card and further confirmed by 16S rRNA sequencing. Whole-genome sequencing (WGS) and antimicrobial resistance (AMR) profiling were performed to identify resistance genes and virulence factors. For genetic analysis, both Illumina short-read and Nanopore long-read sequencing were used, followed by hybrid assembly for enhanced genome resolution. Plasmid and resistance gene profiles were determined using AMRFinder and PlasmidFinder databases.

**Results:**

A total of three CRKP isolates (designated Kp1, Kp2, and Kp3) were identified. Kp1 and Kp2 belonged to sequence type (ST) ST23 and were genetically near-identical, differing by a single allele, while Kp3 was of a distinct sequence type, ST2096, with 245 allelic differences from Kp1 and Kp2. All isolates were resistant to colistin and carried resistance genes, including *mcr-1* and *bla*_NDM-1,_ *bla*_KPC2_ confirming carbapenem resistance. Efflux pump genes and aminoglycoside resistance genes were also detected, providing a multifaceted defence against antibiotics. Plasmid analysis identified several incompatibility groups (IncFI, IncHI, IncFIB, IncX), indicating the potential for horizontal gene transfer of resistance determinants.

**Conclusion:**

This study highlights the complexity of CRKP outbreaks in neonatal care, with isolates exhibiting resistance mechanisms that complicate treatment. The plasmid profiles suggest these strains are reservoirs for multidrug-resistant genes, emphasizing the need for strict infection control and ongoing genomic surveillance. For neonatal care, these resistance challenges increase the risk of treatment failures and mortality, underscoring the importance of enhanced infection prevention and novel therapeutic strategies.

## 1 Introduction

Carbapenem-resistant *Enterobacterales* (CRE), including *Klebsiella pneumoniae*, a non-motile Gram-negative opportunistic pathogen, are major pathogens responsible for severe nosocomial infections worldwide, accounting for approximately 10% of cases (Karampatakis et al., 2023; Cai et al., 2024). These infections carry high mortality rates, particularly among vulnerable populations (Yoo et al., 2023). Mortality rates from CRE infections can exceed 40%-50% in some high-risk groups (Logan and Weinstein, 2017), with *K. pneumoniae* (CRKP) frequently implicated in outbreaks in neonatal intensive care units (NICUs) (Hu et al., 2023). The prevalence of CRKP infection in neonates about 0.3% was reported (Hu et al., 2023).The prevalence of CRE varies widely, with certain regions such as Southeast Asia (Singh et al., 2021), the Mediterranean, and parts of Latin America reporting higher rates, reflecting significant regional and hospital-level burdens (Ma et al., 2023). The first case of CRE in China was documented in 2007 (Xiao et al., 2008, pp. 2004–2005), with resistance rates rising from 0% and 0.7% in 2004 to 1.0% and 13.4% in 2014 (Hu et al., 2016). CRE cases have now been identified in almost every province across China, with their rapid spread largely attributed to the clonal and plasmid-mediated transmission of carbapenem-resistant clinical strains (Zhang et al., 2017). These pathogens often exhibit multi-drug resistance, limiting treatment options to a small number of antibiotics; fewer than 15% of CRE infections respond to common antibiotics, and resistance levels continue to increase globally (Antimicrobial Resistance Collaborators, 2022; Li et al., 2023). The World Health Organization (WHO) recognized CR-KP as a critical priority for new drug development in 2017 due to its extensive resistance to antibiotics, including carbapenems, which are often considered last-resort treatments for multidrug-resistant and extended included in the 2024 list also. underscoring the urgency to address their rapid spread and the challenges they pose in healthcare settings (WHO bacterial priority pathogens list 2024: Bacterial pathogens of public health importance to guide research, development and strategies to prevent and control antimicrobial resistance, n.d). *Klebsiella pneumoniae*, a notable species within CR, is known to cause various healthcare-associated infections, including bloodstream infections, pneumonia, and urinary tract infections (Cai et al., 2024). Neonates are particularly susceptible due to their immature immune systems, extended hospital stays, and exposure to invasive procedures, which collectively increase the risk of nosocomial infections (Tzialla et al., 2024). Carbapenem and cephalosporin antibiotics are commonly used against *K. pneumoniae* infections; however, carbapenem-resistant strains, especially those with additional resistance mechanisms, pose serious therapeutic challenges (Li et al., 2023). Colistin is now a last-resort treatment for multidrug-resistant Gram-negative infections; however, its effectiveness has been undermined by resistance due to the plasmid-mediated mcr-1 gene (Mondal et al., 2024). Since its discovery, studies have identified various mcr gene variants, spanning from *mcr-1* to *mcr-10* (Shen et al., 2022). Eravacycline, a newer tetracycline-class antibiotic that blocks bacterial protein synthesis, offers another option, though resistance is increasingly documented (LaPlante et al., 2022). Resistance in CRKP is often mediated by multiple resistance genes and mechanisms, including *bla*_NDM-1_, *mcr-1*, and *bla*_KPC-2_ (Rodrigues et al., 2024). The *bla*NDM-1 gene encodes a metallo-beta-lactamase that hydrolyses carbapenem antibiotics, such as imipenem and meropenem, rendering them ineffective. The *mcr-1* gene encodes a phosphoethanolamine transferase enzyme, which modifies the lipid A component of the bacterial membrane to confer resistance against colistin. Meanwhile, the *bla*KPC-2 gene encodes a carbapenemase that deactivates carbapenems (Patil et al., 2019). These genes are frequently carried on plasmids, facilitating horizontal gene transfer and rapid spread within healthcare environments. This study aimed to investigate the genomic characteristics and resistance mechanisms of CRKP isolates from a neonatal outbreak, focusing on transmission dynamics and infection control. In this report, we describe a nosocomial outbreak in a neonatology care unit involving CRKP isolates carrying *bla*_NDM-1_, *mcr-1*, and *bla*_KPC-2_ from November to December 2023. These isolates exhibited resistance to both colistin and tetracycline-eravacycline. Next-generation sequencing analysis revealed large multidrug-resistant plasmids harbouring these resistance genes, underscoring the potential for widespread dissemination and highlighting the urgent need for stringent infection control measures. This outbreak represents one of the first reported cases in this setting, emphasizing the importance of monitoring and controlling the spread of these resistance determinants in high-risk healthcare environments.

## 2 Methods

### 2.1 Study setting and case definitions

This study reports an outbreak of CRKP in a 5-bed neonatal intensive care unit (NICU) within a 200-bed tertiary care hospital. The outbreak occurred between November and December 2023, affecting multiple neonates admitted to the NICU. Standard infection control protocols in the NICU included monthly surveillance cultures (nasopharyngeal, oropharyngeal, and rectal swabs) tested for carbapenem-resistant organisms (CROs) and other multidrug-resistant pathogens, including *Staphylococcus aureus*, *Enterococcus faecium*, and Gram-negative bacteria producing extended-spectrum β-lactamases (ESBLs) or carbapenemases (NDM). Prior to this outbreak, no CRKP isolates had been detected in this unit.

### 2.2 Sample collection and microbiological testing

CRKP isolates were obtained from blood and nasal swabs of affected patients in the NICU to identify and characterize the bacterial strains. Initial bacterial identification and antibiotic susceptibility testing were performed on all isolates using the Vitek^®^2 automated system with an N-395 card (bioMérieux). This system provides rapid identification by analysing biochemical reactions, including tests such as the oxidase, catalase, indole production, and carbohydrate fermentation profiles, alongside growth characteristics, which help differentiate bacterial species. The identification process was further confirmed by 16S rRNA sequencing, with sequences analysed using a gene bank database. Antibiotic susceptibility profiling was analysed as per Clinical and Laboratory Standards Institute (CLSI) M100 guidelines (M100Ed34, n.d). Carbapenem resistance was confirmed through meropenem gradient strip testing (E-test), which determines the minimum inhibitory concentration (MIC) of meropenem for each isolate, indicating the level of resistance. To delve further into the genetic mechanisms underlying carbapenem resistance, whole-genome sequencing (WGS) was conducted on each CRKP isolate. WGS allowed for precise identification of carbapenemase genes, such as *bla*NDM, *bla*KPC, and *bla*OXA, as well as other resistance-associated genes and virulence factors. The data obtained from WGS enabled a comprehensive understanding of the genetic elements contributing to carbapenem resistance, as outlined in the methodology section below.

### 2.3 Screening protocols for rectal and environmental cultures

To monitor the spread of CRKP within the NICU, rectal swabs were taken from all patients and incubated in brain heart infusion broth (Thermo Fisher Scientific) containing 16 mg/L amoxicillin for 24 hours at 35°C. Cultures were then plated on selective media, Brilliance CRE agar (Thermo Fisher Scientific) and CHROMID ESBL agar (bioMérieux), and incubated at 35°C for 48 hours. Environmental samples were obtained from high-contact surfaces within the NICU when ongoing transmission was suspected, cultured on 5% sheep blood agar (BA) and MacConkey agar, and incubated at 35°C for 48 hours.

### 2.4 Antibiotic susceptibility testing

AST was conducted on CRKP isolates using the Vitek^®^2 (card N395), gradient strip tests, and the broth microdilution (BMD) method. Minimum inhibitory concentrations (MICs) for meropenem, imipenem, ampicillin/sulbactam, and tigecycline were determined by the gradient strip method (bioMérieux). For these assays, 0.5 McFarland suspensions were prepared from fresh cultures on BA, then inoculated on Mueller Hinton agar (MH, Mediaproducts, Groningen, Netherlands) and incubated at 35°C for 16–20 hours. Additional testing for minocycline and eravacycline was performed using the BMD method following CLSI guidelines (M100Ed34, n.d). MICs were visually assessed as the lowest concentration with no visible bacterial growth after incubation.

### 2.5 Whole-genome sequencing

Genomic DNA was extracted from CRKP isolates using the Ultraclean Microbial DNA Isolation Kit (MO BIO Laboratories). DNA concentration was measured using a Qubit 2.0 fluorometer (Life Technologies), and libraries were prepared using the Nextera XT v2 kit. Illumina short-read sequencing was performed on the MiSeq platform, generating paired-end reads of 300bp. *De novo* assembly was conducted using CLC Genomics Workbench (QIAGEN), with quality trimming set to Q≥20 and a word size of 29.

### 2.6 Nanopore long-read sequencing and hybrid assembly

For isolates requiring additional genetic analysis, high-molecular-weight DNA was prepared following Oxford Nanopore’s protocol SQK-LSK109 and sequenced using the MinION platform (Oxford Nanopore Technologies). Hybrid genome assemblies were created using both Nanopore and Illumina data via Unicycler v0.4.4, with annotation performed by Prokka v1.14.6. Assemblies with ≥95% sequence identity were considered highly similar.

### 2.7 Antimicrobial resistance gene and plasmid analysis

To profile antimicrobial resistance determinants and identify plasmid replicons, assembled sequences were analysed using AMRFinder (v3.8.4), IslandViewer4, and Plasmid Finder (v2.0.2) databases. AMR Finder thresholds were set to 90% identity with a minimum gene length of 60%, while Plasmid Finder used a 95% identity threshold. These profiles were imported into BioNumerics software for analysis.

## 3 Results

### 3.1 Antimicrobial susceptibility testing of CRE *Klebsiella pneumoniae* isolates

A total of three isolates were confirmed as CRKP denoted as (Kp1, Kp2, and Kp3). The antimicrobial susceptibility testing (AST) results for the three CRKP isolates (Kp1, Kp2, and Kp3) are summarized in **Table 1**. Whole genome sequencing (WGS) revealed that isolates Kp1 and Kp2 were genetically nearly identical, while Kp3 exhibited a distinct genetic profile that belonged to ST2096. Kp1 showed minimal inhibitory concentrations (MICs) below the Clinical and Laboratory Standards Institute (CLSI) breakpoints for most antibiotics, except for tigecycline, where it exhibited intermediate resistance. In contrast, Kp2 demonstrated MICs exceeding CLSI breakpoints for carbapenems and ampicillin/sulbactam, while also showing high resistance levels to several tetracyclines, including eravacycline. Additionally, all isolates were resistant to the colistin. Notably, low MICs for minocycline were observed in both Kp1 and Kp2 isolates. For reference, the MICs of ATCC25922 were tested, yielding results consistent with established reference MICs as reported by EUCAST and CLSI (**Table 1**).

**Table 1:** Antimicrobial Susceptibility and Resistance Gene Profiles of *Klebsiella pneumoniae* Isolates, ICU outbreak November December -2023.

Isolates	Days of Admission	Date	ST Type	AST (AMP/AMX/CAZ/TCG/SXT/TOB/GEN/ERA/TET/MINO/CT)	Beta-lactamase Genes	Non-Beta-lactamase Genes
KP1	5 Days	05-Nov-23	ST23	R/R/R/S/R/R/R/R/R/S/R	*bla*_SHV-11_, *bla*_NDM-1_, *bla*_OXA-10,_ *bla*_KPC-2_	*tet*(x3), *sul*2, *ant*(2”)-la, *Acr*AB-TolC *aph*(3”)-lb, *aph*(3’)-la, *aph*(6)-ld, *ant*(3”)-lla, mph(E), *mcr-1.1*
KP2	10 Days	29-Nov-23	ST23	R/R/R/S/R/R/R/R/R/S/R	*bla*_OXA-10,_ *bla*_KPC-2,_ *bla*_NDM-1_	aph(3”)-lb, *aph*(3’)-la, *aph*(6)-ld, ant(3”)-lla, *mph*(E), *msr*, *mcr-1.1*, *Acr*AB-TolC fosA3
KP3	16 Days	15-Dec-23	ST2096	R/R/R/R/R/R/R/R/R/R/R	*bla*_OXA-10_, *bla*_NDM-1_, *bla*_KPC-2_	*aph*(3’)-la, *aph*(6)-ld, *ant*(3”)-lla, *mcr-1.1*, *fos*A3
ATCC25922	-	-		S/S/S/S/S/S/S/S/S/S/S/S/S	-	–

R, Resistant; S, Sensitive; AMP, Ampicillin; AMX, Amoxicillin; CAZ, Ceftazidime; TCG, Tigecycline; SXT, Trimethoprim-Sulfamethoxazole (also known as Co-trimoxazole); TOB, Tobramycin; GEN, Gentamicin; ERA, Erythromycin; TET, Tetracycline; MINO, Minocycline; CT, Colistin.

### 3.2 Genomic analysis of the outbreak *Klebsiella pneumoniae* isolates

WGS analysis classified Kp1 and Kp2 as belonging to multilocus sequence typing (MLST) sequence type ST23, while Kp3 was categorized under ST2096. The close genetic relationship between Kp1 and Kp2 was highlighted by a single allele difference, whereas Kp3 presented 245 allelic differences from Kp1 and Kp2. Analysis using AMRFinder identified several intrinsic chromosomal resistance genes in Kp1 and Kp2, including *bla*_KPC-2_ and *bla*_SHV-11_. Kp2 also contained the fosfomycin resistance gene fosA3, which was absent in Kp1 (**Table 1**).

### 3.3 Analysis of multidrug resistance genome

**Figure 1:**
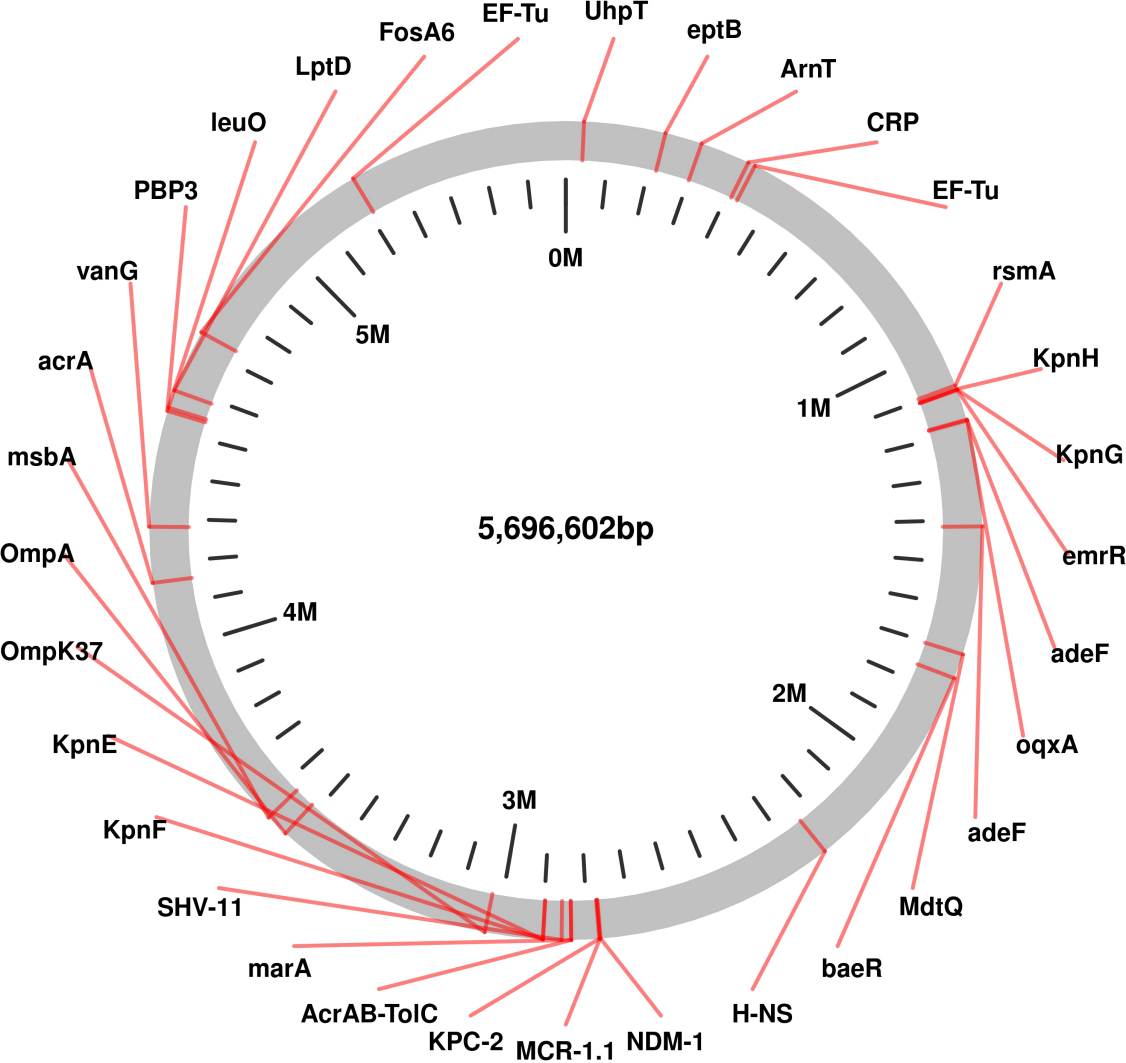
Genomic Circular Map highlighting of Resistance and Virulence Genes in Carbapenem-Resistant *Klebsiella pneumoniae* Isolates Kp-1 recovered from NICU.

**Figure 2:**
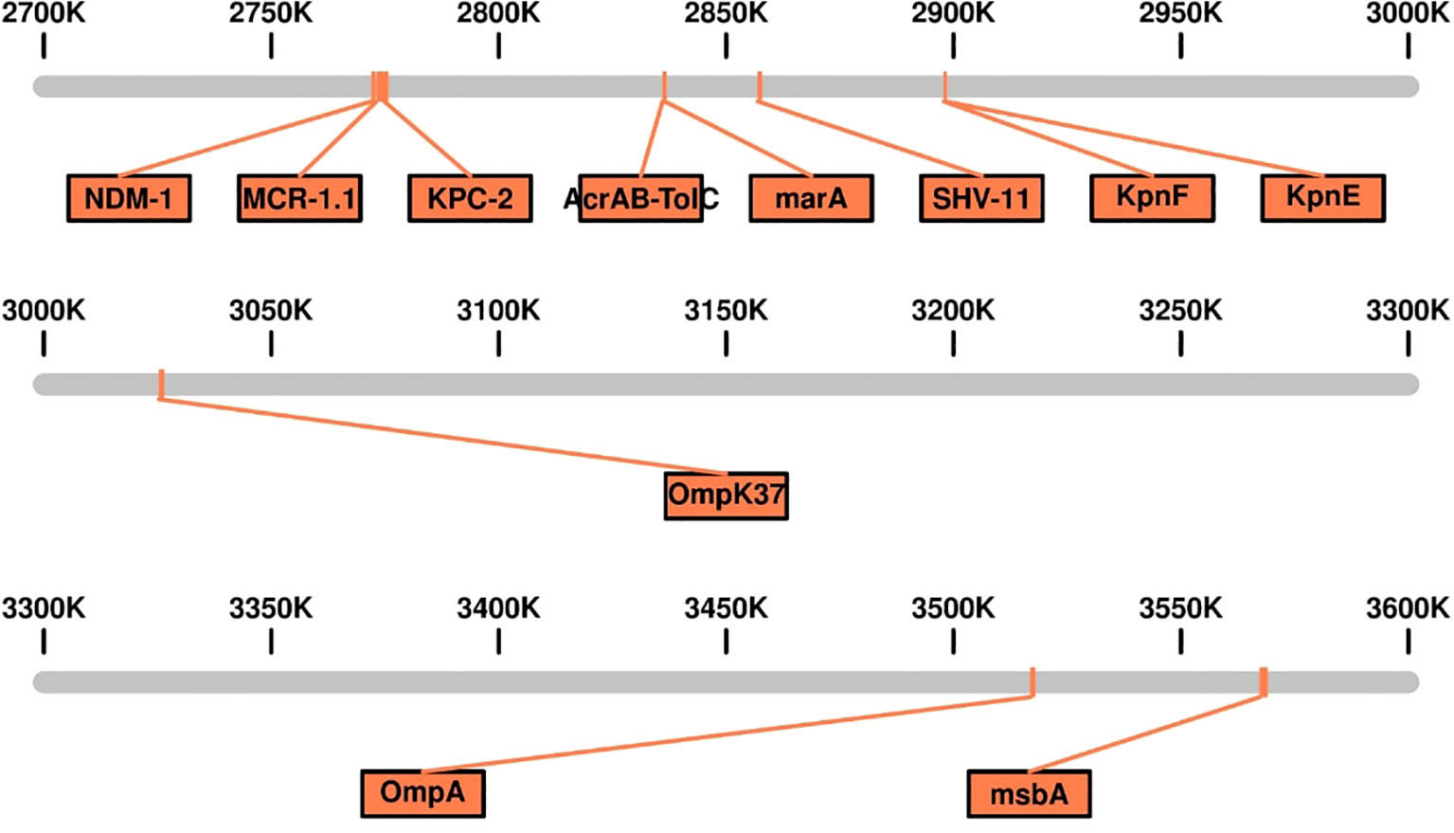
Linear genomic Map of the Genetic Island Containing Multidrug-Resistant (MDR) Genes in Carbapenem-Resistant *Klebsiella pneumoniae* Isolates Kp-1.

The genomic analysis of this multidrug-resistant isolate KP-1 reveals a complex profile of resistance genes distributed across its 5,696,602 bp genome (**Figure 1**). This isolate harbours an array of genes conferring resistance to multiple antibiotic classes, with some genes organized in clusters while others are scattered throughout the genome. Key beta-lactam resistance genes, including NDM-1 and KPC-2, are present, indicating carbapenem resistance (**Figure 2**). NDM-1 (New Delhi metallo-β-lactamase) and KPC-2 (*Klebsiella pneumoniae* carbapenemase) are potent enzymes capable of hydrolyzing a wide range of beta-lactams, including carbapenems, which are typically last-resort antibiotics. The SHV-11 gene is also detected and is associated with resistance to penicillin, potentially enhancing beta-lactam resistance when expressed at high levels. Colistin resistance is suggested by the presence of the mcr-1 gene, which encodes a phosphoethanolamine transferase. This enzyme modifies the lipid A component of lipopolysaccharides, reducing the binding affinity of colistin, a polymyxin antibiotic often used as a last line of defence against multidrug-resistant Gram-negative infections. The genome also includes several genes linked to efflux pumps, such as *acr*A, *msb*A, *Kpn*E, *Kpn*F, *oqx*A, *ade*F, and *Mdt*Q. These efflux pumps actively expel antibiotics from the bacterial cell, reducing intracellular drug concentrations. In particular, the *Acr*AB-TolC efflux pump, indicated by the presence of *acr*A, is significant for its ability to expel a variety of antibiotics, including fluoroquinolones, tetracyclines, and chloramphenicol. Aminoglycoside resistance is conferred by genes such as *aph*(3”)-lb, *aph*(3’)-la, and *aph*(6)-ld, which enzymatically modify aminoglycoside antibiotics, preventing their binding to bacterial ribosomes and protecting the bacteria from the bactericidal effects of drugs like gentamicin, tobramycin, and amikacin. Additionally, regulatory genes such as *mar*A, *bae*R, and *emr*R are present, known to control the expression of various efflux pumps and other resistance mechanisms, often in response to environmental stressors like antibiotic exposure. For instance, MarA (multiple antibiotic resistance regulator) can upregulate the AcrAB-TolC pump, further enhancing the bacterium’s resistance capacity. The H-NS protein, a histone-like nucleoid structuring protein, may contribute by silencing foreign DNA, potentially influencing the expression of resistance genes acquired via horizontal gene transfer. Other genes, such as *Fos*A6, *Uhp*T, and *van*G, further enhance the bacterium’s resistance profile. *Fos*A6 inactivates fosfomycin, while *Uhp*T contributes to resistance through sugar-phosphate transport. *van*G is associated with vancomycin resistance, a trait uncommon in Gram-negative bacteria but potentially contributing to overall survival and resistance. Lipid A modification genes, such as *ept*B and *Arn*T, play roles in resisting cationic antimicrobial peptides, which are part of the host immune response. The genome also encodes various outer membrane proteins and porins, such as OmpA, OmpK37, and LptD. These proteins can affect the membrane permeability to antibiotics. Modifications or downregulation of these porins often reduce antibiotic influx, adding a layer of defence. Overall, this genome analysis highlights a highly resistant bacterial isolate equipped with multiple mechanisms that confer resistance to various antibiotic classes, including beta-lactams, colistin, aminoglycosides, and fosfomycin. The combination of efflux pumps, resistance enzymes, regulatory genes, and membrane proteins creates a robust defence system, underscoring the challenges in treating infections caused by such multidrug-resistant organisms. Plasmid analysis found plasmid present carries Multi drug genes belonging to the Incompatibility groups (Inc) IncFI, IncHI, IncFIB, IncFIS, IncA/C, IncX, IncR, IncI1, and IncQ. Furthermore, the plasmid MOB-subgroup was determined. We found a major subfamily in common which was MOBP: P12, P31, P51 and P131 followed by MOBH: H11, H121; MOBF includes F11, F12 while MOBQ subfamily includes Q11. The PBRT and MOB typing results show that the CRKP are key reservoirs for the diverse group of plasmids which are responsible for the dissemination of drug resistance determinants between other species.

## 4 Discussion

Prior to identifying the CRKP outbreak, preventive measures had already been instituted in the ICU due to a separate outbreak involving non-resistant *K. pneumoniae* strains, including Kp1. These measures included enhanced disinfection protocols, routine patient screening via rectal cultures for all ICU admissions, and environmental monitoring of shared medical devices. After identifying the index patient for the CRE outbreak, infection prevention protocols were intensified, including patient isolation, rigorous cleaning, and intensive hand hygiene training for healthcare personnel. Following the identification of a second CRE-positive patient, additional measures such as a temporary admission halt and thorough disinfection of the ICU were implemented. The emergence of CRKP poses significant challenges in clinical settings, particularly in intensive care units (ICUs) (Li et al., 2024). Our study provided a detailed examination of the resistance mechanisms and genetic characteristics of three carbapenem-resistant *Klebsiella pneumoniae* isolates, offering insights into the epidemiology of this pathogen and important implications for infection control. The AST results revealed a concerning resistance profile, particularly in isolates Kp1 and Kp2, which exhibited high levels of resistance to carbapenems and substantial resistance to other antibiotic classes. The presence of the resistance genes *bla*KPC-2 and *bla*SHV-11 in these isolates underscores their role in mediating resistance (Jin et al., 2024), consistent with findings from global studies that link KPC-type β-lactamases to increased treatment failures in CRKP infections (Sahoo et al., 2024; Cuzon et al., 2010). Similar to reports from studies in the United States (Brennan-Krohn et al., 2022) and Europe (Perez et al., 2016), the detection of these genes highlights a critical barrier in managing infections due to their association with decreased therapeutic options. Notably, low MICs for minocycline were observed in Kp1 and Kp2, suggesting a potential therapeutic alternative for these multidrug-resistant isolates, a finding also supported by studies from Asia that have highlighted minocycline’s efficacy against some CRE strains (Sheu et al., 2019).

The genomic analysis further revealed that Kp1 and Kp2 were closely related, suggesting clonal transmission within the healthcare environment. Identification of the sequence type ST258 in these isolates aligns with global epidemiological data indicating ST258 as a predominant sequence type responsible for outbreaks of multidrug-resistant *K. pneumoniae* worldwide, particularly in North America, Europe, and parts of Asia (Snitkin et al., 2012; Bowers et al., 2015). The distinct genetic profile of Kp3, classified as ST2096, points to the genetic diversity of CRKP in clinical settings, emphasizing the need for continuous genomic surveillance to track the emergence and spread of diverse resistance mechanisms. This diversity, observed in studies from India, China, the Middle East, and Africa, highlights the adaptability of *K. pneumoniae* in acquiring resistance elements from different genetic backgrounds, underscoring the challenge of controlling CRKP (Wyres et al., 2020; Hao et al., 2021; Shankar et al., 2022).

Plasmid analysis provided additional insights into the genetic basis of resistance in these isolates. The detection of multiple resistance genes on plasmids, especially in pKp2, reveals the complex nature of resistance in CRKP. High sequence similarity between plasmids pKp2 and pKp3, along with the presence of integrons, indicates that horizontal gene transfer is likely facilitating the dissemination of resistance traits within the ICU (Realegeno et al., 2021). Of particular concern is the potential for plasmid-mediated interspecies gene transfer, which could amplify the spread of resistance beyond *K. pneumoniae* to other pathogenic species commonly found in the NICU, such as *Enterococcus* spp., *Pseudomonas aeruginosa*, and *Acinetobacter baumannii*. Integrons, which can capture and integrate resistance genes, enhance this process by facilitating the horizontal transfer of these genes across species boundaries (Yao et al., 2024). This finding aligns with reports from other regions, such as Southern Europe and Southeast Asia (Tseng et al., 2023), where integron-associated resistance gene clusters have been implicated in the rapid spread of multidrug-resistant *K. pneumoniae*. The ability of these plasmids to mediate gene transfer to non-*Klebsiella* species significantly complicates infection control measures, as it could create new reservoirs of resistance in the healthcare environment (Ndlovu et al., 2023). The identification of virulence-associated genes on the plasmid adds another layer of complexity to clinical management, as it suggests that these isolates not only exhibit robust antibiotic resistance but also have an increased potential for virulence, a factor noted in studies from China (Ma et al., 2024). During the outbreak, neonates received combination antibiotic therapy, including colistin and tigecycline, with limited success. Patient isolation and rigorous disinfection protocols were implemented, reducing cross-transmission rates within a short period.

In response to the CRE outbreak, infection control measures, including proactive screening and rigorous hygiene protocols, were implemented to control the spread of these multidrug-resistant isolates. This approach is consistent with infection control strategies recommended globally, which emphasize proactive containment to manage the spread of CRKP in healthcare facilities. However, despite these measures, the persistence of *K. pneumoniae* cultures among patients and environmental surfaces highlights ongoing challenges in eradicating potential reservoirs. Similar challenges have been documented in other studies from high-incidence regions like South America and the Middle East, indicating that continuous and adaptive infection control efforts are essential to mitigate the risk of future outbreaks and prevent *K. pneumoniae* from establishing itself as a persistent pathogen in healthcare settings. Environmental contamination, including on surfaces such as incubators, feeding tubes, ventilators, and medical equipment, serves as a persistent reservoir for multidrug-resistant organisms (MDROs). These surfaces are in frequent contact with colonized or infected patients, facilitating the survival and spread of resistant bacteria, often despite routine cleaning procedures. While this study provides critical insights into the dynamics of CRKP outbreaks in the ICU setting, there are several areas for future investigation. The relatively small sample size of isolates (n=3) limits the generalizability of the findings, and further studies involving a larger number of clinical isolates from different healthcare settings would help confirm the observed trends. Moreover, our plasmid analysis provided important information, but more detailed investigations, including whole-genome sequencing of a broader range of CRKP isolates, could provide deeper insights into the role of horizontal gene transfer in the spread of resistance and virulence traits. The findings on minocycline susceptibility, while promising, would benefit from further clinical evaluation to determine its true therapeutic potential in the management of CRKP infections. Finally, long-term surveillance and continued implementation of infection control measures will be essential to understanding the trajectory of CRKP in ICUs and developing more effective strategies for managing these challenging infections.

## 5 Conclusion

Our study highlights the genetic characteristics and resistance mechanisms of CRKP, underscoring significant infection control challenges. The presence of *bla*_KPC-2_ and *bla*_NDM-1_ co-existence of *mcr-1* genes confirms high resistance levels in these isolates, consistent with global findings linking these genes to treatment failures. Clonal spread within the healthcare setting and the presence of virulence genes on resistance plasmids emphasize the need for strict monitoring and enhanced infection control measures. To mitigate the risk of future outbreaks in NICUs, we recommend proactive surveillance, stringent hygiene protocols, cohorting strategies, and antimicrobial stewardship followed by education and training. Our findings underscore the critical importance of ongoing surveillance, proactive screening, and rigorous hygiene protocols to prevent future outbreaks of multidrug-resistant *K. pneumoniae*.

## Data Availability

Publicly available datasets were analyzed in this study. This data can be found here: NCBI/PRJNA1192565.
